# Performance evaluation of a large-scale thermal power plant based on the best industrial practices

**DOI:** 10.1038/s41598-020-77802-8

**Published:** 2020-11-26

**Authors:** Yousef S. H. Najjar, Amer Abu-Shamleh

**Affiliations:** Mechanical Engineering Department, Jordan University of Science and Technology, Irbid, Jordan

**Keywords:** Diesel fuel, Natural gas

## Abstract

The aim of this study is to assess and evaluate the performance of a large-scale thermal power plant (TPP). The performance rating was conducted in compliance with the statistical principles. The data for this analysis were obtained for a TPP with an installed capacity of 375 MW during a span of 8 years (2010–2017). Four parameters were used to evaluate the performance of the TPP including the availability, the reliability, the capacity factor, and the thermal efficiency. These parameters were calculated using a set of equations and then compared to the international best practices and target values. The results indicate that approximately 91% of the expected capacity was available throughout the studied period against the industry best practice of 95%. However, the average TPP’s reliability was found to be approximately 95% against the target value of 99.9%. Furthermore, the average capacity factor throughout the studied period is 70% as against the international value of 40–80%. Moreover, the thermal efficiency of the TPP is 40% against the target value of 49%. Due to the outage hours and malfunctions, the power losses throughout the studied period reached 846 MW. Overall, the analysis indicates that the studied TPP is not within the scope of the best industrial practices.

## Introduction

The gas turbine (GT), also known as the combustion turbine, is a rotary motor that removes energy from a hot gas flow generated in a stream of compressed air by combustion of gas or fuel oil. The GT has a radial or axial flow air compressor mechanically connected with an upstream turbine and combustion chamber. Energy is released by the mixing and ignition of compressed air into the combustion chamber (combustor)^1^. Energy is generated in shaft power by GTs and used to power generators and other machinery. GTs have been recognized as prime movers for reliable baseload applications and they are being increasingly used world wide^2,3^.

GTs have earned a privileged position among other electrical generation technologies due to its high efficiency and reliability, particularly when incorporated with combined cycle^4^. GTs are also known for their flexibility and regular availability^4^. However, the performance of the GT is influenced by both the efficiency of components and the turbine inlet temperature^5,6^. In addition, the operation of GTs and combined-cycle operations is predominantly affected by the long-term operation. In addition, the output of the gas-turbine engine has relatively poor performance at part-load, and power output (${P}_{\mathrm{out}}$) deteriorates during hot seasons^7,8^. Najjar et al.^9^ studied the performance diagnostics and the degradation of the GT cycle using actual data obtained from a combined-cycle power plant throughout 2 years of operation. It has been concluded that the degradation of the GT increases with temperature and load over time. In this context, the performance ratings of thermal power plants (TPPs) including reliability, availability, capacity factor, and efficiency, are expected to flocculate with the operation time.

CCGT is one type of power plant that directs the exhaust gas of the GT over a heat exchanger which generates steam at various levels of pressure^10^. The performance analysis of the combined-cycle gas turbine (CCGT) focuses mainly on evaluating the power efficiency of the plant^11^. The efficiency of a plant has a definite economic significance because heat inputs are the energy that is to be purchased at high temperatures, and net energy production is the return on the energy that is purchased. The GT running at lower inlet temperatures of the turbine produces low performance thus, lower efficiency^12^. The lower efficiency of the GT means that low ${P}_{\mathrm{out}}$ is produced. Several factors affecting the efficiency of TPPs in general, these include age, fuel type, capacity factor, and heat sink system^13^.

Sabouhi et al.^14^, investigated the reliability of a CCGT power plant using a developed model. The analysis involved modeling both GT and steam turbine power plants from an engineering system perspective, which provided the necessary data to estimate the reliability of the CCGT. Overall, the results point out to the most important components that help in selecting convenient strategies for the CCGT power plants^15^. On the other hand, Kolawole et al.^16^ studied the availability, reliability, and capacity factor of a power generating plant using its historical data. It has been concluded that plant unavailability, the grid constraints, and gas restriction prevented the power plant from running at the maximum continuous rating. However, they suggested that in order to enhance the power supply, there is a demand for better maintenance, adequate gas supply, and the examination of the distribution and transmission units.

Lamfon et al.^17^ studied the performance of a GT with a capacity of 23.7 MW operated at an ambient temperature between 30 and 45 °C. They reported an 11% improvement in the net ${P}_{\mathrm{out}}$ when the inlet of the GT engine is provided with cold air. However, an increase of 11% in the net ${P}_{\mathrm{out}}$ is also reported at an ambient temperature of 30 °C. This was based on the International Standard Organization (ISO) rated condition along with a 2% increase in $\eta$ with a 2% decrease in specific fuel consumption. However, Ameri et al.^18^ investigated the variation of ambient temperature on the ${P}_{\mathrm{out}}$. It has been pointed out that if the ambient temperature drops from 34.2 °C to the ISO-rated condition an average increase in the ${P}_{\mathrm{out}}$ by as much as 11.3% can be achieved. It has also been reported that the ${P}_{\mathrm{out}}$ efficiency decreased by 0.74% when the ambient air temperature increased by 1 °C. In another similar study, Mohanty et al.^19^ concluded that the net ${P}_{\mathrm{out}}$ decrease by 10% when the inlet air temperature increases by 30% from the ISO-rated conditions. Nevertheless, for small-scale GTs, this incline in the net ${P}_{\mathrm{out}}$ can be much greater. In addition, the study showed that raising the ambient temperature by 1 °C would result in about a 1% drop in the rated capacity of the GT.

Given the important role of energy in economic development in a country and the anticipated substantial future requirements, it is a key choice to conserve energy and make productive usage. The aim of this paper is to report on an evaluation method to assess the performance of a TPP in terms of availability, reliability, capacity factor, and thermal efficiency, for a period of 8 years (from 2010 to 2017). The proposed evaluation methodology will help in the performance diagnostics of similar power plants. Moreover, this evaluation is useful for linking maintenance to the overall performance of the power plant, thus, helping in maintenance scheduling.

## Power plant description

The plant is situated in the northern part of Jordan, 70 km from the capital Amman. The region where the plant is located has a warm climate with average ambient temperature and relative humidity of 18 °C and 48% respectively. The plant is located within a rural region approximately 832 m above sea level.

**Figure 1:**
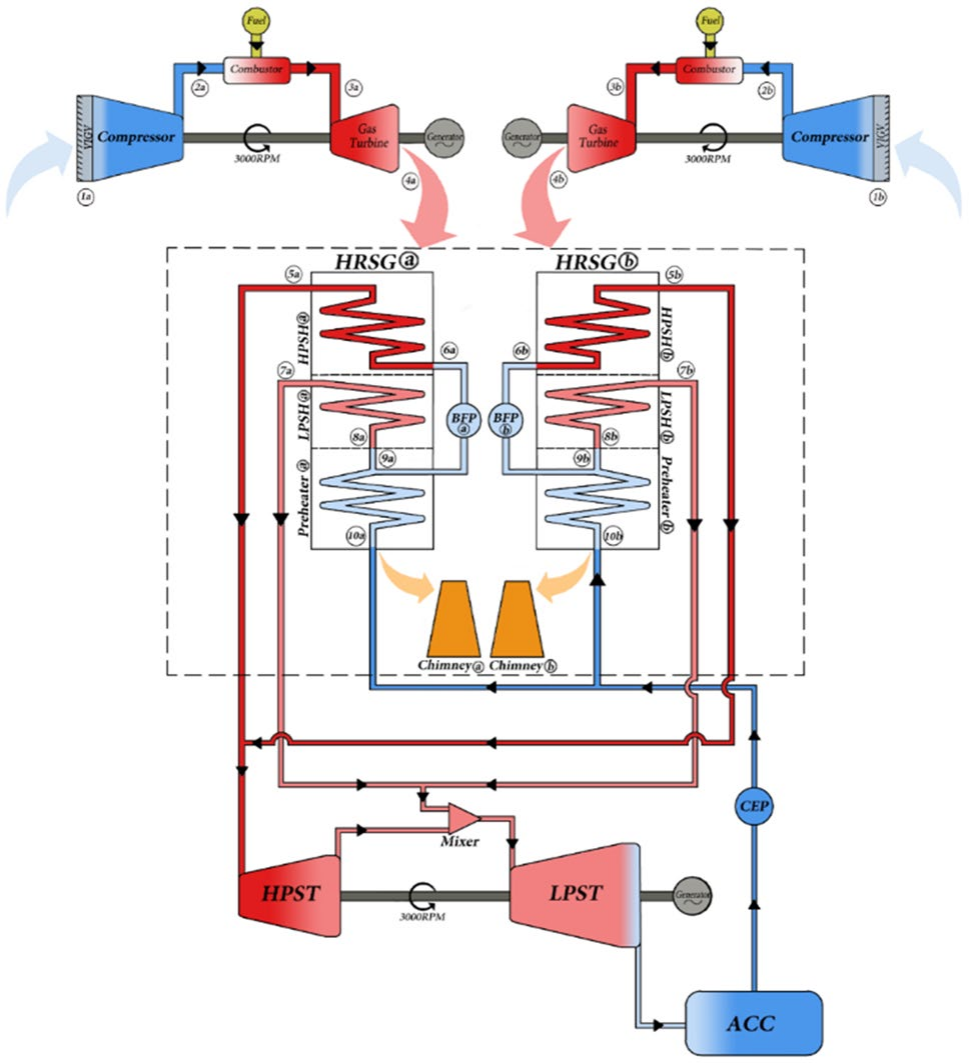
A schematic of process flow throughout a CCGT. Reprinted from Sustainable Energy Technologies and Assessments, 37, Najjar, Y. S. H., Alalul, O. F. A. & Abu-Shamleh, Degradation analysis of a combined cycle heat recovery steam generator under full and part load conditions. Sustainable Energy Technologies and Assessments, 100,587, 2020, with permission from Elsevier.

The power plant consists of two simple-cycle GTs with a rated capacity of 30 MW each and a 297 MW CCGT comprised of two GTs which are nominally rated at 100 MW each and one ST with a rated capacity of 97 MW. The plant utilizes two types of fuels namely, diesel oil and natural gas and uses dry cooling fans for cooling. Figure 1 shows a schematic diagram of a CCGT.

## Data collection

The data of the power plant were obtained for a period of 8 years (2010–2017) from CEGCO annual reports^20,21^. Throughout the studied period, several major malfunctions occurred to the power plant. Though minor failures were also observed during the extended period, the major failures were observed for the years 2010, 2012, and 2013. These malfunctions affected the overall performance of the power plant and caused some of the generation units to be out of service for several days. The total number of days the malfunctioned units were out of service throughout the studied period is 206. Table 1 summarizes the major failures the power plant was exposed to during the period of the study.

**Table 1:** A summary of the major malfunctions that the power plant was exposed to during the studied period.

Year	Incident	Cause	Period	No. of days	Comments
2010	One GT was out of service	The teeth of the high-speed gear were partially broken due to an emergency failure	(12/2–23/3) and (14–25/7)	51	The GT was returned to service at full capacity on 25/7/2010
	One GT was out of service	The side of the turbine was twisted and separated due to a failure in the accessory gear coupling	(3/2–12/2)	10	A new accessory gear coupling was installed to fix the failure
2012	One GT was out of service	Failure of one of the turbine shafts and the existence of a problem in the unit torque convertor	(15/6–11/7)	27	The torque converter was replaced by a new one
2013	One GT was out of service	Failure in the generator of the unit	(23/4–18/8)	118	–

The specific data that was used to evaluate the power plant performance includes, total installed capacity (MW), power output (MW), generated electrical energy (${E}_{g}$) (MWh), running hours (h), heat supply (MW), and heat rate (HR) (kJ/kWh). Table 2 shows the data of the power plant throughout the studied period.

**Table 2:** The data of the power plant across the studied period.

Year	Total installed capacity	Power output (MW)	Generated electrical energy (MWh)	Running hours (h)	Heat supply (MW)	Heat rate (kJ/kWh)
2010	357	279.95	2,197,800	7850.71	707.84	8941
2011	357	242.16	2,013,600	8314.99	598.23	8731
2012	357	244.86	1,994,400	8145.05	600.59	8674
2013	357	224.81	1,620,000	7205.98	565.14	8900
2014	357	258.02	2,108,400	8171.33	632.88	8830
2015	357	255.70	2,041,900	7985.62	656.31	9240
2016	357	240.09	1,963,100	8176.58	613.10	9193
2017	357	245.97	2,105,800	8561.15	623.50	9124.9

## Performance analysis

The performance evaluation was carried out based on statistical principles. The evaluation was carried out based on four key parameters namely, availability, reliability, capacity factor, and thermal efficiency. These parameters are calculated based on the data of the power plant and then compared to the best industrial practices and target values.

The forced outage factor (FOF) and the planned outage factor (POF) were both used to calculate the annual running hours of the TPP by subtracting the number of hours per year from both. FOF is defined as the shutdown of the plant as a result of undesired occurrences, whereas POF is defined as the prescheduled shutdown as for routine maintenance^22^. Equations 1–9 were used to calculate the identified 4 parameters. The calculated FOF and POF are presented in Table 3.

**Table 3:** The outages factors of the TPP.

Year	Forced outage hours (h)	Planned outage hours (h)	Forced outage factor (%)	Planned outage factor (%)
2010	548.38	360.91	6.26	4.12
2011	204.98	240.02	2.34	2.74
2012	430.12	184.84	4.91	2.11
2013	1267.57	286.45	14.47	3.27
2014	122.64	466.03	1.4	5.32
2015	109.50	664.88	1.25	7.59
2016	127.90	455.52	1.46	5.2
2017	14.02	184.84	0.16	2.11

The running hours of the power plant per each year can be calculated using the following equation:

1 $$Running\,hours= 24 \left[\frac{h}{day}\right]\times 365\left[\frac{days}{year}\right]-(FOH \left[h\right]-POH \left[h\right])$$

where $FOH$ is the forced outage hours. and $POH$ is the planned outage hours.

The forced outage factor can be calculated as follows:

2 $$FOF\,[\%] = \frac{FOH\,[h] }{24 \left[\frac{h}{day}\right]\times 365\left[\frac{days}{year}\right]}\times 100$$

The planned outage factor can be calculated as follows:

3 $$POF\,[\%] = \frac{POH\,[h] }{24 \left[\frac{h}{day}\right]\times 365\left[\frac{days}{year}\right]}\times 100$$

The power output of the plant in MW was obtained as follows:

4 $${P}_{out}\,[MW]= \frac{{E}_{g}\,[MWh]}{Running\,hours[h]}$$

The availability of the power plant is calculated using the following equation:

5 $$Availability\,[\%] = \frac{Running\,hours[h] }{24 \left[\frac{h}{day}\right]365\left[\frac{days}{year}\right]}\times 100$$

The reliability of the power plant is calculated using the following equation:

6 $$Reliability\, \left[\%\right]= 1- \frac{FOH[h] }{24 \left[\frac{h}{day}\right]\times 365\left[\frac{days}{year}\right]-(FOH \left[h\right]+POH \left[h\right])}\times 100$$

The capacity factor of the power plant is calculated using the following equation:

7 $$Capacity\,Factor \left[\%\right]= \frac{{E}_{g} }{24 \left[\frac{h}{day}\right]\times 365\left[\frac{days}{year}\right] \times Total\,Installed\,Capacity\,[MW]}\times 100$$

The thermal efficiency (${\eta }_{th}$) of the power plant throughout the studied period is calculated as follows:

8 $${\eta }_{th}\,[\%] = \frac{{P}_{out}[MW] }{Heat\,Supply\,[MW]} \times 100$$

Finally, the HR of the power plant can be calculated as follows:

9 $$HR \left[\frac{kJ}{kWh}\right]= \frac{3600}{{\eta }_{th}}$$

## Results and discussion

**Figure 2:**
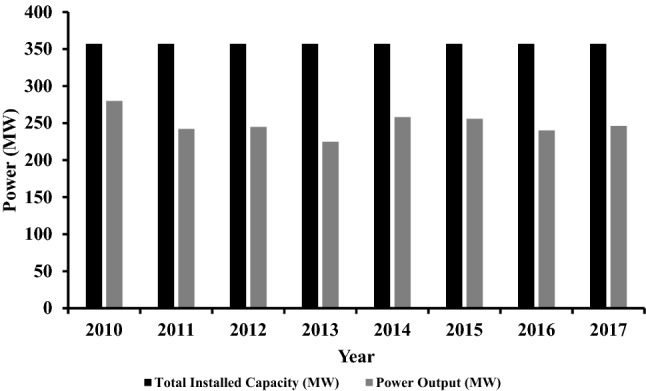
The generated power by the TPP throughout (2010–2017).

The calculated values for each rating parameter across the studied period are shown in Table 4. These values are fluctuated based on the performance of the TPP during the years. Ideally, the total generated power of the TPP during the 8 years can be calculated by multiplying the total installed capacity by the number of the years. In this case, the total generated power in MW should be 2856 MW, however, the actual generated power is less than that, due to the TPP being operated at part load conditions, along with other technical reasons including outage hours and malfunctions. The actual power generated throughout the studied period is approximately 1991.6 MW, meaning that 864.4 MW was lost. Overall, this operating loss does not mean that the plant is not achieving its purpose as most of the power plants are expected to have such huge losses during the years. In this context, the performance analysis is used to decide whether these losses are significant or not. Figure 2 illustrates the actual power generated to the installed generated power.

**Table 4:** The calculated performance parameters of the TPP.

Year	Availability (%)	Reliability (%)	Capacity factor (%)	Thermal efficiency (%)
2010	89.62	93.01	76.7	39.55
2011	94.92	97.53	70.3	40.48
2012	92.98	94.72	69.6	40.77
2013	82.26	82.41	56.6	39.78
2014	93.28	98.50	73.6	40.77
2015	91.16	98.63	71.3	38.96
2016	93.34	98.44	68.5	39.16
2017	97.73	99.84	73.5	39.45

**Figure 3:**
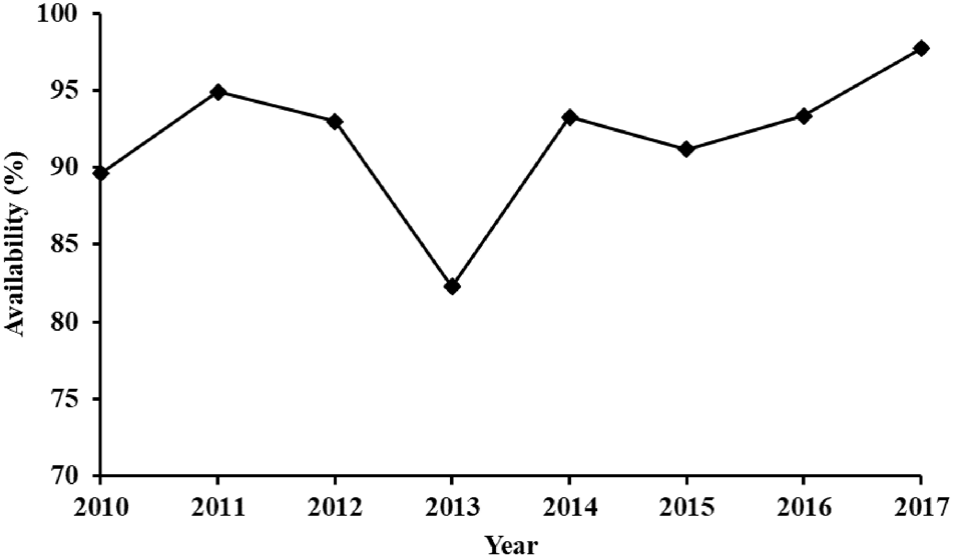
The availability factor of the TPP throughout (2010–2017).

The availability factor of a power plant is the amount of time that it is able to produce electricity over a certain period, divided by the amount of time in the period^23^. The availability factor depends on the operation of the power plant, the fuel type, and the design of the plant. In this study, the availability of the studied TPP varies from 82.3 to 97.7%. Considering that the best industrial practices designate that GTs have a relatively high availability factor of 95%^24^, the TPP, on average, is not within the target value. Figure 3 shows the variation of the availability factor over the studied period.

**Figure 4:**
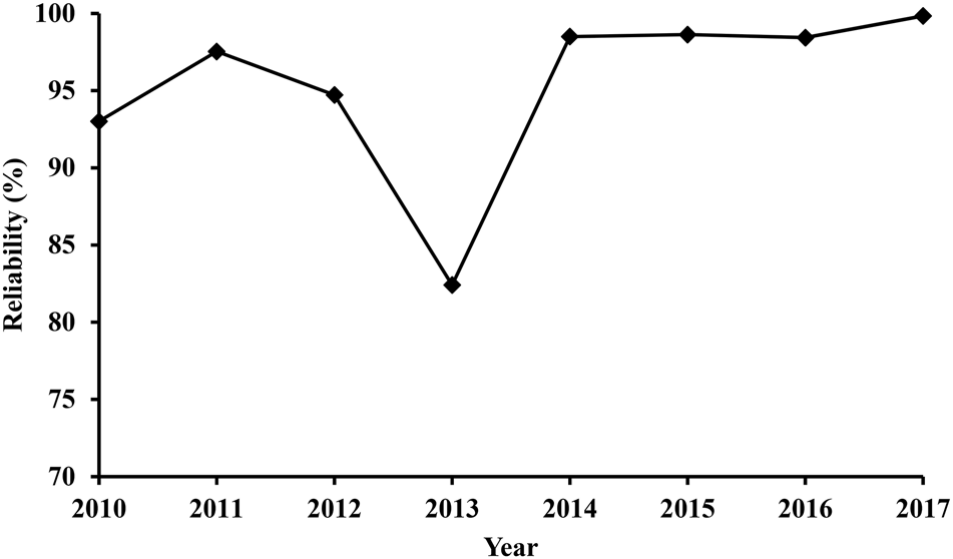
The reliability factor of the TPP throughout (2010–2017).

The reliability analysis is an important step in the evaluation of CCGTs and plays a significant role in the operation of the plant in terms of maintenance scheduling^25^. However, the reliability factor of a power plant is mainly dependent upon the FOH and can be calculated by dividing the FOH by the actual time of operation. Over the studied period, the reliability of the TPP ranged between approximately 82.4% and 99.8% as shown in Fig. 4. The lowest value has been obtained for the year 2013 due to one GT being out of service for a relatively large amount of time as mentioned in Table 1. Nevertheless, the TPP’s average reliability was found to be 95.4%. Since there is no mentioning of the best industrial range of reliability for CCGTs in the literature, this percentage was compared to the starting reliability value of 99.9%^26^. This means that the reliability of the power plant is not within the best industrial practices. The high average percentage obtained can be attributed to the annual percentage of hours due to forced outage and the planned outage is around 4%.

**Figure 5:**
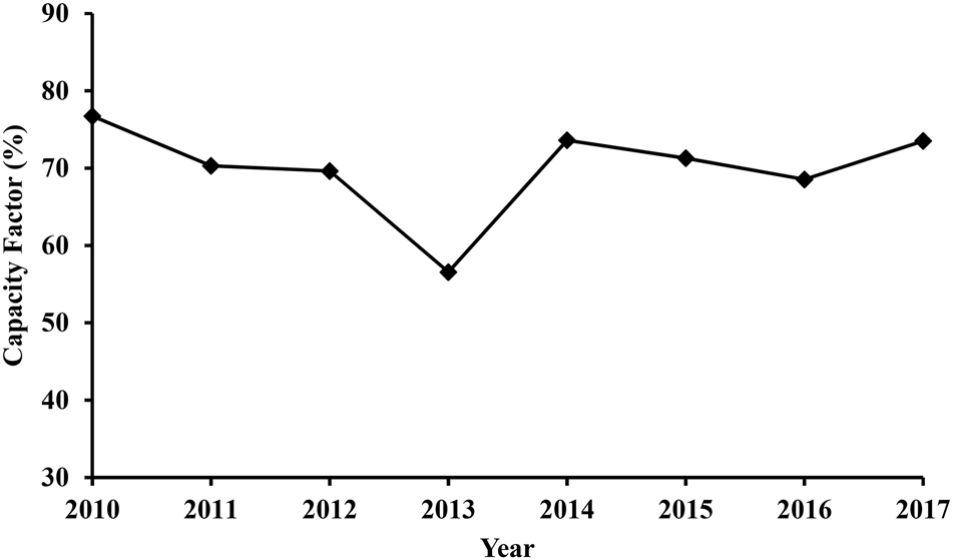
The capacity factor of the TPP throughout (2010–2017).

The capacity factor of a power plant is essentially a measure of its overall utilization^27^. For a power plant, the capacity factor can be calculated by dividing the actual electricity produced by its maximum possible electricity output throughout a certain period of time^28^. The average capacity factor of the TPP over the studied period is 70% with a minimum value of 65.6% obtained for 2013 and a maximum value of 76% for the year 2010. The flocculation of the capacity factor can be attributed to the age of the plant, outages, operation and maintenance, and weather conditions. Based on the international values, the average capacity factor of a power plant within a specific period should not be less than 65%^26^, meaning the power plant is within the normal ranges. Figure 5 shows the graph of the capacity factor throughout the studied years.

**Figure 6:**
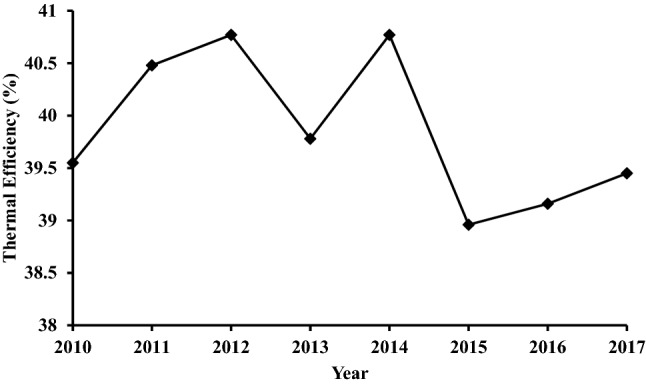
The thermal efficiency of the TPP throughout (2010–2017).

The thermal efficiency of the power plant is defined as the power output of the plant divided by the heat supplied. The thermal efficiency mainly depends on the heat value of the fuel used and the temperature. As the inlet temperature of the turbine increases, the thermal efficiency increases^29^. The thermal efficiency of the studied TPP throughout (2010–2017) is shown in Fig. 6. The average thermal efficiency of the TPP is approximately 39.9%. The maximum obtained value is approximately 40.8% for both 2012 and 2016, while the minimum value is approximately 39.8% for the year 2013. Although the large malfunctions that occurred in 2013 do not directly affect the thermal efficiency, the cut in the fuel supply as a result does. Since the power plant is composed of two units (simple and combined), the obtained efficiency was compared to the target value of 49%. This value is the average between the approximate thermal efficiency of simple cycle gas power plants which is in the range of 35–42%^30^, and that average value for CCGTs which is 60%^31^.

GTs are designed for standard air conditions. Nevertheless, the operating times are much greater at off-design conditions than at site conditions. When a GT operates at site environmental conditions different from the ISO, a distinction can be found between the actual power produced by a GT and the design-rated power marked on the GT. A comprehensive study and recording of operating data have shown that the ambient temperature is related to direct degradation of the GT capacity^32^. The GT loses 1% of its ${\eta }_{th}$ and 1.47 MW of gross capacity for each 1 °C increase in the ambient temperature above the ISO limit.

The GT's power generation output is dependent on the turbine inlet temperature. The temperature of the turbine inlet plays an important role in the performance of a cycle system. The efficiency of the component and the working temperature of the turbine affect the GT performance. The overall efficiency of the CCGT is mainly dependent on the compressor pressure ratio and turbine inlet temperature^33^. The key performance parameters are summarized and shown in Table 5. These parameters are compared to the industry's best practices and target values.

**Table 5:** Summary of the values of the key performance parameters against the best industrial practices.

Performance parameter	Obtained value (%)	Industry best practice (%)	Deviation (%)	References
Availability	91	95	− 4	^24^
Reliability	95	99.9	− 4.5	^26^
Capacity factor	70	50–80	+ 5	^26^
Thermal efficiency	40	49	− 9	^30,31^

## Conclusions

In this study, a large-scale TPP was evaluated based on statistical principles. The evaluation was based on collected data from an actual power plant for a period of 8 years. The main performance parameters that were studied include availability, reliability, capacity factor, and thermal efficiency. These parameters were compared to the best industrial practices and target values. The average availability and reliability of the TPP throughout the studied period were found to be approximately 91% and 95% respectively. In addition, the average capacity factor and thermal efficiency over the studied period were found to be 70% and 40% respectively. The power losses out of the total generated electrical power over the studied period reached 864.4 MW. Several major malfunctions were reported over the studied period which caused the TPP to be out of service for a number of days. These malfunctions are linked to the overall performance of the TPP and the evaluated parameters. Overall, the analysis indicates that the plant is not within the context of the best industrial practices. Moreover, the evaluation methodology followed in this research can be useful in building maintenance schedules for such plants.

## Recommendations

The CCGT efficiency and reliability can be improved considerably. The following are suggested ways to improve the performance of such a plant:

1. Increasing the inlet temperature of the turbine, taking into consideration the building material of the turbine can withstand high temperatures or by replacing the current parts with others that can withstand high temperatures. For 56 °C, the work output rises by around 10%, and the overall efficiency also increases by 1.5%^34^.
2. Suitable maintenance and cleaning of the inlet filters of the compressors. Dirty and poorly maintained filters cause significant efficiency loss as the compressor blades suffer clogging and damage. In addition, the pressure drop can be caused by dirty filters. Thus, Regular part checks and cleaning will boost plant performance and reliability.

## List of symbols

CCGT: Combined-cycle gas turbine
*E*_*G*_: Generated electrical energy (MWh)
FOF: Forced outage factor (%)
FOH: Forced outage hour (h)
GT: Gas turbine
HR: Heat rate (kJ/kWh)
ISO: International Organization for Standardization
POF: Planned outage factor (%)
POH: Planned outage hour (h)
*P*_out_: Power output (MW)
TPP: Thermal power plant

## Greek letter

*η*_*th*_: Thermal efficiency (%)

## Subscripts

*a*: Air
1: Air compressor inlet
2: Combustor outlet
3: Gas turbine inlet
4: Gas turbine outlet
5: Inlet of high-pressure heat recovery steam generator
6: Outlet of high-pressure heat recovery steam generator
7: Inlet of low-pressure heat recovery steam generator
8: Outlet of low-pressure heat recovery steam generator
9: Inlet of heat recovery steam generator preheater
10: Outlet of heat recovery steam generator preheater
